# Odontoid fractures: impact of age and comorbidities on surgical decision making

**DOI:** 10.1186/s12893-020-00893-7

**Published:** 2020-10-14

**Authors:** Syed Ali Mujtaba Rizvi, Eirik Helseth, Pål Rønning, Jalal Mirzamohammadi, Marianne Efskind Harr, Tor Brommeland, Mads Aarhus, Christina Teisner Høstmælingen, Håvard Ølstørn, Pål Nicolay Fougner Rydning, Magnus Mejlænder-Evjensvold, Nils Christian Utheim, Hege Linnerud

**Affiliations:** 1 Faculty of Medicine, University of Oslo, Oslo, Norway; 2 Department of Neurosurgery, Oslo University Hospital, Mailbox 4956, 0424 Oslo, Norway; 3 Department of Neuroradiology, Oslo University Hospital, Oslo, Norway

**Keywords:** Spinal fractures/epidemiology, Odontoid process/injury, Age factors, Comorbidity, Risk factors, Spinal fractures/surgery, Guideline adherence

## Abstract

**Background:**

Surgical fixation is recommended for type II and III odontoid fractures (OFx) with major translation of the odontoid fragment, regardless of the patient’s age, and for all type II OFx in patients aged ≥50 years. The level of compliance with this recommendation is unknown, and our hypothesis is that open surgical fixation is less frequently performed than recommended. We suspect that this discrepancy might be due to the older age and comorbidities among patients with OFx.

**Methods:**

We present a prospective observational cohort study of all patients in the southeastern Norwegian population (3.0 million) diagnosed with a traumatic OFx in the period from 2015 to 2018.

**Results:**

Three hundred thirty-six patients with an OFx were diagnosed, resulting in an overall incidence of 2.8/100000 persons/year. The median age of the patients was 80 years, and 45% were females. According to the Anderson and D’Alonzo classification, the OFx were type II in 199 patients (59%) and type III in 137 patients (41%). The primary fracture treatment was rigid collar alone in 79% of patients and open surgical fixation in 21%. In the multivariate analysis, the following parameters were significantly associated with surgery as the primary treatment: independent living, less serious comorbidities prior to the injury, type II OFx and major sagittal translation of the odontoid fragment. Conversion from external immobilization alone to subsequent open surgical fixation was performed in 10% of patients. Significant differences the in conversion rate were not observed between patients with type II and III fractures. The level of compliance with the treatment recommendations for OFx was low. The main deviation was the underuse of primary surgical fixation for type II OFx. The most common reasons listed for choosing primary external immobilization instead of primary surgical fixation were an older age and comorbidities.

**Conclusion:**

Major comorbidities and an older age appear to be significant factors contributing to physicians’ decision to refrain from the surgical fixation of OFx. Hence, comorbidities and age should be considered for inclusion in the decision tree for the choice of treatment for OFx in future guidelines.

## Background

The incidence of traumatic cervical spine fractures (CS-fx) in the Norwegian population is 15/100,000/year [1]. Twenty percent of these fractures are odontoid fractures (OFx) of cervical vertebra 2 (C2) [2]. The incidence of this injury increases with age, making OFx the most common CS-fx in the elderly population [2–4]. OFx are subdivided into types I, II and III according to the classification proposed by Anderson and D’Alonzo [5]. Type I fractures are very rare and do not require stabilization, while type II and III fractures are common and considered unstable.

A minor proportion of patients with OFx die immediately at the scene of the accident due to severe fracture dislocation with subsequent injury to the upper spinal cord, causing tetraplegia and respiratory arrest [6–8]. However, the majority of patients present with an OFx after trauma with various degrees of neck pain. When left untreated, these patients are at risk of fracture dislocation with secondary spinal cord injury (SCI) or persistent and severe neck pain due to chronic instability/pseudarthrosis. The aims of treatment are to preserve neurological function, provide pain relief and establish bony fusion. A consensus for the management of OFx is currently lacking, and the choice of treatment has mainly been guided by the fracture type (II or III), magnitude and direction of displacement of the OFx fragment, patient age and the knowledge of variations in bony fusion rates after the use of different treatment options [9–11].

Several publications recommend primary surgical fixation for type II and III OFx with major translation of the odontoid fragment, regardless of the patient’s age, and for all type II OFx in patients aged ≥50 years [4, 11–14]. For the remaining patients, primary external immobilization is advised. These previous recommendations do not consider the effects of an older age and comorbidities of the patients, nor the possibility of a stable fibrous union as a satisfactory result of treatment. Recent reports have questioned these previous recommendations and advocate that patient age and comorbidities should be included in future treatment algorithms and that a stable fibrous union should be regarded as an acceptable outcome [15–18].

The recommendation of surgery for all patients aged ≥50 years with OFx type II is a subject of debate, since most of these fractures occur in the elderly with major comorbidities and increased risks of surgical morbidity and mortality [16, 19, 20]. The bony fusion rate of OFx type II in patients aged ≥50 years is higher after surgical fixation than after external immobilization in a rigid collar or Halo-vest for 12 weeks [11, 13, 21]. However, according to recent reports, many patients will exhibit a stable fibrous union of the OFx after 12 weeks of external mobilization, despite the lack of bony fusion [15–18]. This fibrous union is defined by a lack of pathological movement of the odontoid fragment upon dynamic flexion and extension studies performed under fluoroscopic guidance, despite the absence of radiological bony fusion.

The level of compliance with the aforementioned treatment recommendations in our department is unknown. In the current study, we present updated epidemiological data on OFx in a defined population of 3.0 million people. The effects of the fracture type, displacement of the odontoid fragment, patient age and comorbidities on the rate of open surgical fixation are discussed. Our hypothesis is that open surgical fixation is less frequently performed than recommended, and we suspect that this discrepancy might be due to the older age and comorbidities observed in patients with OFx.

## Methods

Oslo University Hospital (OUH) is a level 1 trauma centre located in Oslo, Norway and the only major trauma care facility in the Southeastern Norwegian Regional Health Authority (Norwegian: Helse Sør-Øst RHF). OUH is the only hospital in this region with neurosurgical service. In 2017, the southeastern region of Norway had 2.95 million inhabitants with the following age distribution: 1913884 aged 0–49 years, 381,157 aged 50–59 years, 318,407 aged 60–69 years, 215,141 aged 70–79 years, 97,333 aged 80–89 years and 24,623 aged > 90 years [22]. OUH performs > 95% of the trauma-related neurosurgical procedures in this population, including all surgeries for cervical spine injury. Twenty hospitals within our region with general and/or orthopaedic surgeons and radiological services refer patients with head and cervical spine injuries to OUH. The patients are either admitted to OUH for treatment or are managed locally when the neurosurgical team at OUH has decided that conservative treatment is indicated. We use a rigid collar for external immobilization. Treatment with a Halo-vest was discontinued in our region several years ago.

This study employed a prospective observational cohort design and examined all patients in the southeastern Norwegian population diagnosed with a traumatic OFx in the period from 2015 to 2018. The data were retrieved from our quality control database for traumatic CS-fx in southeastern Norway. In this database, all patients with CS-fx (C0/C1 to C7/Th1) who were diagnosed using cervical CT within our region are prospectively registered. Only patients with an 11-digit unique Norwegian social security number who were living within the region were included. The database was approved by the OUH - Data Protection Officer (PVO) (approval no 2014/12304), generated in Medinsight, and run by the Department of Neurosurgery. The present study, which is based on data extracted from this quality database, was approved by OUH-PVO, and consent was waived by our Institutional Review Board (approval no 18/02167).

For this study, the following data were extracted from the database: date of injury, injury mechanism, gender, age at the time of injury, living status at the time of injury (home – care for self, home – with assistance, or institutionalized), pre-injury American Society of Anesthesiologists (ASA) score [23], type of OFx (types I-III) according to the classifications proposed by Anderson and D’Alonzo [5] and Grauer et al. [24], sagittal displacement of the odontoid fragment (direction and magnitude), presence of other cervical fractures (yes/no), multi-trauma (yes/no), Head Injury Severity Score (HISS) [25], presence of a concomitant thoracolumbar fracture (yes/no), presence of a SCI (yes/no), treatment (external immobilization with rigid collar alone or open surgical fixation), compliance with the previous treatment recommendations for OFx with respect to surgical fixation (yes/no) and reason for non-compliance based on a chart review.

The pre-injury ASA score was defined as follows: 1. a normal healthy patient, 2. a patient with a mild systemic disease, 3. a patient with a severe systemic disease, 4. a patient with severe systemic disease that is a constant threat to life, and 5. a moribund patient who is not expected to survive without surgery.

Previous treatment recommendations for OFx include primary surgical fixation for type II and III fractures with major displacement of the odontoid fragment, regardless of the patient’s age, and for all type II fractures in patients aged ≥50 years. For the remaining patients with OFx, primary external immobilization is recommended.

Major sagittal displacement of the odontoid fragment was defined as a translation ≥5 mm anterior or ≥ 3 mm posterior [13].

Reasons for non-compliance were categorized as follows: surgery was not performed due to an older age and/or comorbidities, the diagnosis was delayed > 12 weeks, or the presence of a non-survivable injury.

Multi-trauma was defined as a simultaneous traumatic brain injury (mild, moderate or severe), and/or radiology-proven (X-ray, CT or ultrasound) injury in one or more of the following regions: face, thoracolumbar spine, thorax, abdomen, pelvic or extremities. Skin injuries were not registered.

### Statistical analysis

Data were summarized as frequencies and means or medians, according to the distribution of the variables. Pearson’s chi-squared test was used to compare differences in frequencies between groups. The Kruskal-Wallis test was used to compare the distributions between multiple groups. A Poisson model was used to investigate the effect of age on the incidence of injury in the different age groups. Both uni- and multivariate logistic regression models were fitted for binary dependent variables. The R package v3.6 was used for statistical analyses. *P*-values less than 0.05 were considered significant.

## Results

**Fig. 1:**
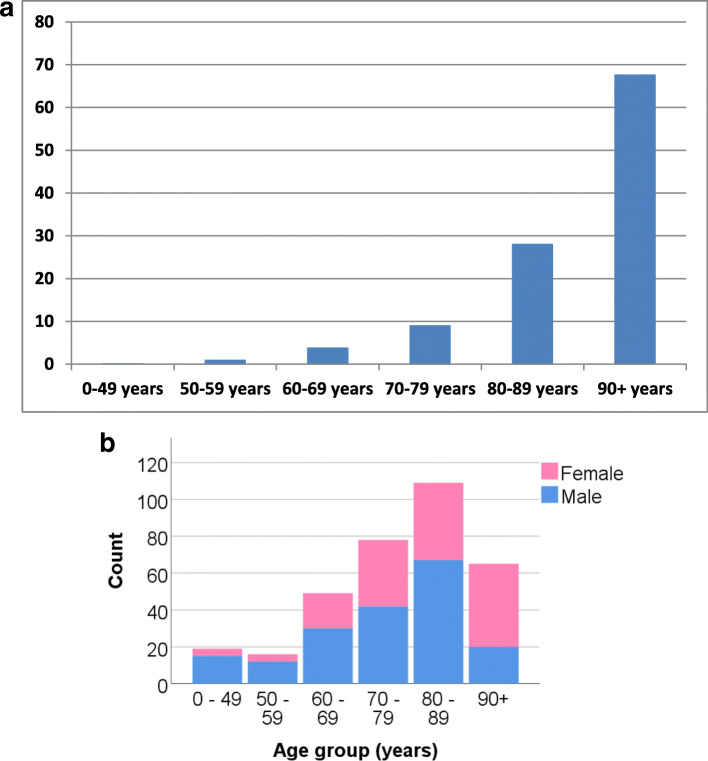
**a**: Age-adjusted incidence rates (x/100000 persons/year). **b**: Number of odontoid fractures in groups stratified according to age and gender

In our defined population of 3.0 million people in southeastern Norway, 336 patients with an OFx were prospectively registered during a 4 year period from 2015 to 2018, resulting in an overall OFx incidence of 2.8/100000 persons/year. However, the incidence in the elderly was considerably higher (Fig. 1a).

Patient characteristics and injury descriptions are presented in Table 1. The median patient age was 80 years (range 6–100 years), and 150/336 (44.6%) were females. The number of OFx increased with age (incidence rate ratio 2.5 for 10 year increments, 95% CI 2.3–2.9, *p* < 0.001). A male predominance was observed in younger patients, while a gradual shift to a female predominance was observed among older patients (Fig. 1b). The OFx were classified as type II fractures in 199 patients (59.2%) and type III fractures in 137 patients (40.8%). Type II OFx were more frequent than type III OFx (*p* < 0.001). Major sagittal translation was observed in 48/196 (24.5%) of patients with a type II OFx and 21/137 (15.3%) of patients with a type III OFx. Translation data were missing for 3 patients (Table 2).

**Table 1:** Patient characteristics

		336 (100%)
Sex	Male	186 (55.4)
	Female	150 (44.6)
Age group (years)	0–49	19 (5.7)
	50–59	16 (4.8)
	60–69	49 (14.6)
	70–79	78 (23.2)
	80–89	109 (32.4)
	90+	65 (19.3)
Pre-injury ASA score^a^	ASA score of 1	33 (9.8)
	ASA score of 2	89 (26.5)
	ASA score of 3	187 (55.7)
	ASA score of 4	26 (7.7)
	Not available	1 (0.3)
Dependence in daily life	Home – independent	220 (65.5)
	Home – with assistance	62 (18.5)
	Institution	53 (15.8)
	Not available	1 (0.3)
Injury mechanism	Fall	288 (85.7)
	Motorized vehicle (MV)	16 (4.8)
	Bicycle	15 (4.5)
	Pedestrian hit by MV or bicycle	5 (1.4)
	Other	12 (3.6)
Odontoid fracture	Type II	199 (59.2)
	Type III	137 (40.8)
Other spine fractures	C1	64 (19.1)
	C0 (occipital condyle)	9 (2.7)
	C3 – Th1	27 (8.1)
	Thoracolumbar	30 (8.9)
Spinal cord injury (SCI)	Odontoid fx	13 (3.9)
	Other cervical fx	1 (0.3)
	Thoracolumbar fx	2 (0.6)
Multi-trauma	Yes	143 (42.6)
	TBI^b^ - Mild	91 (27.1)
	TBI - Moderate	11 (3.3)
	TBI - Severe	9 (2.7)
	Face	29 (8.6)
	Thoracolumbar fx	30 (8.9)
	Thorax	29 (8.6)
	Abdomen	5 (1.5)
	Pelvic	5 (1.5)
	Extremity	27 (8.0)

^a^*ASA* American Society of Anesthesiologists

^b^*TBI* Traumatic Brain Injury

**Table 2:** Uni- and multivariate analyses of variables potentially associated with the use of surgery as the primary treatment

Variable		Primary conservative treatment N (%)	Primary surgery N (%)	OR^a^ (univariate)	OR (multivariate)
Sex	Female	129 (86.0)	21 (14.0)	–	–
	Male	137 (73.7)	49 (26.3)	2.20 (1.26–3.93, *p* = 0.006)	1.44 (0.71–2.95, *p* = 0.311)
Age	Mean (SD^b^)	78.3 (16.1)	71.0 (11.4)	0.97 (0.96–0.99, *p* = 0.001)	1.00 (0.98–1.02, *p* = 0.904)
Functional status	Dependent	110 (95.7)	5 (4.3)	–	–
	Independent	155 (70.5)	65 (29.5)	9.23 (3.95–27.01, *p* < 0.001)	5.86 (2.15–19.08, *p* = 0.001)
ASA^c^ score	ASA score of 1–2	77 (63.6)	44 (36.4)	–	–
	ASA score of 3–4	189 (87.9)	26 (12.1)	0.24 (0.14–0.42, *p* < 0.001)	0.30 (0.13–0.65, *p* = 0.003)
Odontoid fracture type	Type II	136 (68.0)	64 (32.0)	–	–
	Type III	130 (95.6)	6 (4.4)	0.10 (0.04–0.22, *p* < 0.001)	0.08 (0.03–0.19, *p* < 0.001)
Odontoid translation	None-Mild	225 (85.2)	39 (14.8)	–	–
	Major^d^	38 (55.1)	31 (44.9)	4.71 (2.63–8.47, *p* < 0.001)	5.59 (2.70–11.99, *p* < 0.001)

^a^*OR* Odds ratio

^b^*SD* Standard deviation

^c^*ASA* American Society of Anesthesiologists

^d^Major odontoid translation - ≥5 mm anterior or ≥ 3 mm posterior

Pre-injury major comorbidities (ASA ≥3) were present in 213 (63.7%) of the patients, and 115 (34.3%) required assistance with ADL (Table 1). An increasing age was significantly associated with both a greater number of comorbidities (*p* < 0.001) and a need for assistance with ADL (*p* < 0.001). Furthermore, a significant association was observed between a high ASA score and the need for assistance with ADL (*p* = 0.02).

The most common trauma mechanism was falls (85.7%), followed by motorized vehicle accidents (4.8%) and bicycle accidents (4.5%) (Table 1). The fraction of fall-related injuries increased significantly with 10 year increments in age (1.71, 95% CI (1.42–2.08), p < 0.001), from 53% in the group aged < 50 years to 95% in patients aged > 80 years. Multi-trauma was registered in 142 patients (42.6%). SCI due to OFx was present in 13 patients (3.9%).

The primary fracture treatment was a rigid collar alone in 266 patients (79.2%) and open surgical fixation followed by external immobilization in 70 patients (20.8%). In the univariate analysis, the following parameters were significantly associated with the use of surgery as the primary treatment: male sex, young age, independent living, pre-injury ASA score of 1–2, type II OFx and major sagittal translation of the odontoid fragment (Table 2). In the multivariate analysis, the following parameters remained significantly associated with the use of surgery as the primary treatment: independent living, pre-injury ASA score of 1–2, type II OFx and major sagittal translation of the odontoid fragment (Table 2). Male sex and age were not associated with the use of surgery as the primary treatment in the multivariate analysis.

Conversion from external immobilization alone to subsequent open surgical fixation was performed in 26/266 patients (9.8%) due to increased dislocation, severe neck pain hindering mobilization or pseudarthrosis. Treatment conversion was performed in 15/135 (11.1%) of patients with a type II OFx compared with 11/131 (8.4%) of patients with a type III OFx. This difference in conversion between patients with type II and III fractures was insignificant (*p* = 0.618).

Our compliance with the aforementioned previous treatment recommendations for OFx is presented in Table 3 and appears to be low. The three largest patient categories in our series were patients aged ≥50 years with a type II fx (category 1, *n* = 184), patients with a type III fx without major translation (category 2, *n* = 116), and patients with a type III fx with major translation (category 3, *n* = 21). For patients in category 1, the recommended treatment is primary surgical fixation, but this procedure was only performed in 33% of patients. For patients in category 2, the recommended treatment is primary external immobilization, which was performed in 98% of patients. For patients in category 3, the recommended treatment is primary surgical fixation, but it was only performed in 19% of patients. The main deviation from the previous guidelines was the underuse of primary surgical fixation. Based on chart reviews, the reasons listed for choosing primary external immobilization instead of primary surgical fixation included a combination of age and comorbidities in 122 patients, lethal injury in 8 patients and delayed diagnosis in 8 patients (Table 4).

**Table 3:**
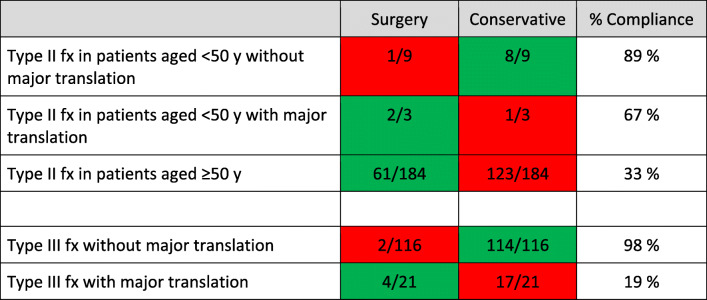
Rate of compliance with published treatment recommendations for odontoid fractures [4, 11–14]. Green indicates compliance and red indicates non-compliance. Translation data were missing for 3 patients who were treated conservatively

**Table 4:** Rate of non-compliance with published treatment recommendations for odontoid fractures, and reasons why primary external immobilization was chosen instead of primary surgical fixation [4, 11–14]

	Reason for non-compliance	N (%)
Compliant		195 (58.0)
Non-compliant	Age/comorbidities	122 (36.3)
	Lethal injury	8 (2.4)
	Delayed diagnosis	8 (2.4)
Missing data		3 (0.9)

## Discussion

As shown in the present study, the estimated incidence of traumatic OFx in the southeastern Norwegian population is 2.8/100000 persons/year. The fractures mainly occurred in elderly patients with significant comorbidities. The primary treatment was external immobilization alone in 79% and open surgical fixation in 21%. In the multivariate analysis, independent living, pre-injury ASA scores of 1–2, type II OFx and major sagittal translation of the odontoid fragment were significantly associated with the use of surgery as the primary treatment. The low rate of surgery is intriguing, since previous reports have documented that the bony fusion rate is higher after surgical fixation than after external immobilization alone.

### Epidemiology

A specific International Classification of Disease (ICD) code is not available for OFx [26]. This fracture shares the ICD-10 code S12.1 with all other forms of C2 fractures. This finding is important, since the majority of previous epidemiological studies report the incidence of all types of C2 fractures and not odontoid fractures exclusively, as they are based on the extraction of ICD codes from registries. A Swedish nationwide registry study conducted from 1997 to 2014 reported that the incidence of C2 fractures increased from 3/100000 persons/year in 1997 to 6/100000 persons/year in 2014 [27]. These numbers are not directly comparable to our results, as the C2 fractures were not sub-typed. Another Swedish regional study estimated the incidence of OFx in the period from 2002 to 2014 to be 3/100000 persons/year [3]. Based on a United States nationwide registry on hospitalized patients for the period from 2000 to 2010, the incidence of C2 fractures is approximately 1/100000 persons/year [28]. A reasonable conclusion would be that the Norwegian incidence of OFx of 2.8/100000 persons/year is within the range of previously published results, although these studies provide a limited basis for comparison.

The median age of our patients was 80 years, and the frequency of OFx increased with increasing age in our study population. This finding is consistent with numerous other reports [3, 27–30]. The reported increase in incidence of OFx and C2 fractures in the geriatric population during the few last decades cannot be explained simply by an extended life expectancy. One can argue that the use of CT instead of plain X-ray as the primary diagnostic tool probably explains phenomenon portion of this increase [27–30].

A slight male predominance (55%) was observed in our patients with OFx. However, this predominance disappeared with increasing age, and among patients aged > 90 years, the number of women with an OFx outnumbered men. This result corresponds to the increasing proportion of women in the older age groups of the population [22]. Similar findings have also been reported by other groups [27, 31].

An increasing age was significantly associated with a greater number of comorbidities, as measured using the pre-injury ASA score, and the need for assistance with ADL. Previous studies have also documented increased numbers of comorbidities among geriatric patients sustaining OFx [32–37]. Age and comorbidities are of course linked to some extent, but we postulate that they act as separate risk factors for OFx. The risk of healthy older people to suffer an OFx is probably lower than older people with comorbidities. Illness often leads to inactivity or a neurological disability, which pre-disposes an individual to osteoporosis and an increased risk of unintended falls [38]. A higher burden of disease will lead to the use of medications in many cases, and several classes of medications are associated with an increased risk of falls in the elderly [39]. The WHO has defined risk factors for falls, including polypharmacy treatment, comorbidities, an age > 80 years, and impaired cognition and vision [40]. The presence of a medical comorbidity may also predispose patients to an increased risk of complications in the setting of fracture care, such as thromboembolic disease, cardiac events and infections. The in-hospital mortality rates in this patient group, regardless of the choice of treatment, have been reported to be as high as 10–25% [33, 35, 37, 41, 42], and 1-year mortality rates are 20–50% [19, 20, 37, 42, 43]. The principal causes of death are related to their comorbidities and not the injury itself for the majority of patients with OFx [19, 20]. This high burden of disease represents a major concern in many of these patients regarding whether they will be able to tolerate general anaesthesia and open surgery.

The most common trauma mechanism among our patients was falls (85.7%), followed by motorized vehicle accidents (4.8%) and bicycle accidents (4.5%) The fraction of fall-related injuries was 53% in the group aged < 50 years and 95% in patients aged > 80 years. Several authors have identified falls as the major trauma mechanism in elderly patients suffering an OFx, particularly falls from a standing height [15, 31, 42, 44–46].

### Fracture morphology

In our cohort of patients with OFx, 59% had type II fractures and 41% had type III fractures. Interestingly, type I fractures were not observed. Our proportion of patients with type II OFx is somewhat lower than the values reported from the UK and Sweden. In a UK study, type II and type III OFx constituted 84 and 16% of the injuries, respectively [31], while a Swedish study reported 1% type I, 69% type II and 29% type III fractures [3]. All studies, including ours, used the Anderson and D’Alonzo classification with the Grauer modification.

SCI secondary to dislocation of the odontoid fragment was observed in 13 patients (3.9%) in our series. Other researchers have reported a rate of SCI in patients with OFx or all C2 fractures ranging from 2 to 6% [27, 37, 42, 47]. The number of SCIs after OFx may be higher than reported, since a severe SCI at the C2 level may be fatal at the scene of the accident [6–8, 48].

### Treatment

The main treatment goals for patients with OFx are to preserve the neurological function, relieve pain and establish a stable fusion. To date, no real consensus has been achieved and class I evidence for the management of these fractures is lacking [9, 11]. Historically, “stable fusion” has been rated synonymously with bony fusion. Recent publications have proposed that age and comorbidities should be emphasized as independent variables in treatment algorithms for OFx and that a fibrous union might be a valid result of treatment in these patients [16, 33, 41, 49–52].

External immobilization is typically achieved using a Halo-vest or stiff neck collar. A meta-analysis of 12 studies (714 fractures) comparing the Halo-vest with collar immobilization identified an equivalent rate of non-union between the treatment groups, but the number of complications more than doubled in patients treated with Halo-vests [53]. Thus, when choosing conservative treatment, a reasonable approach is to primarily use a rigid neck collar. When surgery is chosen, the evidence is in favour of performing a posterior screw fixation if the goal is bony fusion, which has historically been the definition of a successful treatment [10].

Most likely, a large proportion of elderly patients and patients with comorbidities presenting with OFx have not been included in randomized control trials (RCTs) comparing conservative and surgical treatment for OFx due to the high surgical risks. In non-randomized cohort studies, a high potential for selection bias exists because surgeons tend to select healthier patients for surgery. This bias in the published literature should always be considered when evaluating reported outcomes after the treatment of odontoid fractures in the geriatric population.

### Rate of surgery in southeastern Norway

Despite the evidence for a superior bony fusion rate after the surgical fixation of OFx, we have suspected that the rate of surgery for these fractures in our department is low. We also postulated that the reasons for this potentially low rate of surgery were the older age and comorbidities among the patients. These hypotheses were verified in this study, as we chose surgery as the primary treatment for 21% of the total patients, and surgery was chosen more often for patients with type II OFx than type III OFx. The largest group in which we refrained from treating the patients according to previous recommendations was patients with a type II OFx aged > 50 years. The low use of primary surgical fixation in our study was clearly associated with an older age and comorbidities. The main concern with this low rate of surgery is a potentially high rate of treatment failure after external immobilization alone. In our series, the failure of external immobilization alone leading to subsequent open surgical fixation occurred in 9.8% of patients. Primary external immobilization of type II OFx was not associated with a higher rate of conversion to open surgical fixation compared to type III OFx (11.1% versus 8.4%). A 9.8% failure rate may be considered high. However, 90.2% of the patients selected for external immobilization were successfully treated, most of whom were high risk candidates for surgery due to age and severe comorbidities.

The high rate of clinically successful treatment with external immobilization alone, despite the expected low rate of bony fusion, indicates that stable fibrous union is an acceptable outcome.

Reported rates of surgery for OFx vary considerably, but many series present a low surgery rate (12–28%) in elderly patients [37, 41, 42]. A few publications present a higher surgery rate, with 46–53% of patients in all age groups undergoing surgery for type II OFx and 13–19% of patients undergoing surgery for type III OFx [3, 31]. Studies reporting on all C2 fractures in the elderly in the United States show a surgery rate of 10–16% [54, 55]. Because the majority of C2 fractures in the elderly are type II OFx, the findings imply a low surgical treatment rate in the elderly with this type of fracture.

### Limitations of the study

This study only presents epidemiological data and a description of our current treatment practice for patients with OFx. Based on the results of the present study, we are unable to conclusively determine whether this low rate of surgery is acceptable or represents a suboptimal practice.

We hope to answer this important question with our large observational cohort study of > 500 consecutive patients with OFx who were treated from 2009 to 2017. The data from the follow-up of this cohort are now being processed and we believe that the results will provide important insights into management of these fractures.

## Conclusions

In southeastern Norway, 21% of patients with OFx were treated with primary open surgical fixation. Major comorbidities (ASA ≥3) were present prior to trauma in approximately 2/3 of the patients, 1/3 needed assistance with ADL, and more than half of the patients were ≥ 80 years of age.

Major comorbidities and an older age appear to be significant factors contributing to the decision to refrain from the surgical fixation of OFx. Hence, comorbidities and age should probably be considered for inclusion in the decision tree for the choice of treatment for OFx in future guidelines.

## Data Availability

Our database contains sensible data which might provide insight in clinical and personnel information about our patients and lead to identification of patients. According to organization restrictions and regulations the data cannot be made public available. Data are however available from the authors upon reasonable request and with permission of the Data Protection Officer at OUH.

## Abbreviations

CS-fx: Cervical spine fractures
OFx: Odontoid fractures
C2: Cervical vertebra 2
SCI: Spinal cord injury
OUH: Oslo University Hospital
ASA: American Society of Anesthesiologist
HISS: Head Injury Severity Scale
ADL: Activities of daily living
ICD: International Classification of Disease
